# Frequency dependence and the predictability of evolution in a changing environment

**DOI:** 10.1002/evl3.266

**Published:** 2021-12-20

**Authors:** Luis‐Miguel Chevin, Zachariah Gompert, Patrik Nosil

**Affiliations:** ^1^ CEFE, Univ Montpellier, CNRS, EPHE, IRD Montpellier 34090 France; ^2^ Department of Biology Utah State University Logan Utah 84322 USA

**Keywords:** Changing environment, chaos, fluctuating selection, frequency dependence, predictability

## Abstract

Frequency‐dependent (FD) selection, whereby fitness and selection depend on the genetic or phenotypic composition of the population, arises in numerous ecological contexts (competition, mate choice, crypsis, mimicry, etc.) and can strongly impact evolutionary dynamics. In particular, negative frequency‐dependent selection (NFDS) is well known for its ability to potentially maintain stable polymorphisms, but it has also been invoked as a source of persistent, predictable frequency fluctuations. However, the conditions under which such fluctuations persist are not entirely clear. In particular, previous work rarely considered that FD is unlikely to be the sole driver of evolutionary dynamics when it occurs, because most environments are not static but instead change dynamically over time. Here, we investigate how FD interacts with a temporally fluctuating environment to shape the dynamics of population genetic change. We show that a simple metric introduced by Lewontin, the slope of frequency change against frequency near equilibrium, works as a key criterion for distinguishing microevolutionary outcomes, even in a changing environment. When this slope *D* is between 0 and –2 (consistent with the empirical examples we review), substantial fluctuations would not persist on their own in a large population occupying a constant environment, but they can still be maintained indefinitely as quasi‐cycles fueled by environmental noise or genetic drift. However, such moderate NFDS buffers and temporally shifts evolutionary responses to periodic environments (e.g., seasonality). Stronger FD, with slope *D* < –2, can produce self‐sustained cycles that may overwhelm responses to a changing environment, or even chaos that fundamentally limits predictability. This diversity of expected outcomes, together with the empirical evidence for both FD and environment‐dependent selection, suggests that the interplay of internal dynamics with external forcing should be investigated more systematically to reach a better understanding and prediction of evolution.

What causes variation in evolutionary trajectories, and to what extent can we predict these trajectories over meaningful timescales? Beyond randomness (drift and contingency of mutation) and uncertainty (measurement error) reducing the predictability of evolution (Crow and Kimura 1970; Gould 1989; Lenormand et al. 2009; Blount et al. 2018; Nosil et al. 2020), an important question in many long‐term studies of natural populations is: What causes temporal variation in natural selection? And can we predict how these causes vary over time, to predict in turn variation in selection and evolutionary change? Numerous investigations of natural selection over repeated years in the wild have shown that the direction and/or strength of selection may vary over time (Reimchen 1995; Grant and Grant 2002; Reimchen and Nosil 2002; Siepielski et al. 2009; Bell 2010; Morrissey and Hadfield 2012; Rouzic et al. 2015; Nosil et al. 2018; de Villemereuil et al. 2020). However, the reason for this variation is less often demonstrated, not to mention directly quantified, for instance by regressing selection gradients, optimum phenotypes, or selection coefficients, against putatively causal environmental variables (Wade and Kalisz 1990; MacColl 2011; Chevin et al. 2015; Siepielski et al. 2017; Gompert 2021). Yet the search for these causes is a necessary step toward understanding and projecting evolutionary change.

In particular, a critical question that has yet received little attention is: When natural selection varies over time, is it because a variable external biotic or abiotic environment acts as a forcing factor on the population, as suspected for instance for seasonal cycles in allelic frequency in fruit flies (Bergland et al. 2014), or adaptation to climate change across a range of organisms (Hoffmann and Sgro 2011)? Or is it instead because ecological feedbacks cause natural selection to depend on the current state of the population, leading to internally driven dynamics, as also clearly established in natura (Sinervo and Lively 1996; Olendorf et al. 2006; Rouzic et al. 2015; Chouteau et al. 2016; Bolnick and Stutz 2017; Nosil et al. 2018; Goldberg et al. 2020)? Different traditions in evolutionary biology (both theoretical and empirical) tend to favor one or the other explanation, sometimes based on prior knowledge and experience of a study system, but often also on the preference and scientific background of the authors.

On the one hand, a large body of literature focuses on adaptation to changing environments and its interplay with extinction risk, in particular with respect to global climate change and environmental degradation (Lynch and Lande 1993; Bürger and Lynch 1995; Chevin et al. 2010; Hoffmann and Sgro 2011; Kopp and Matuszewski 2014). In this context, natural selection and its variation over time are generally assumed to result from change in the external environment. This is envisioned to cause the displacement of an optimum phenotype, which the population then has to track by evolution, phenotypic plasticity, or their combination, as demonstrated empirically in some case studies (Vedder et al. 2013; Chevin et al. 2015; Gamelon et al. 2018; de Villemereuil et al. 2020; Gauzere et al. 2020), and invoked verbally in many others. Empirical work in this field often aims at testing or applying predictions from an abundant theoretical literature on adaptation to a moving optimum (Lynch and Lande 1993; Bürger and Lynch 1995; Chevin et al. 2010; reviewed by Kopp and Matuszewski 2014).

On the other hand, studies that focus on eco‐evolutionary feedbacks (Hendry 2016; Lion 2018; Govaert et al. 2019) or genetic conflicts (Hurst et al. 1996; Chapman et al. 2003) tend to emphasize situations where the evolutionary dynamics of a population are mostly driven by its own evolution. This includes a large body of empirical work on the maintenance of visible polymorphisms (Sinervo and Lively 1996; Halkka et al. 2001; Oxford 2005; Nosil et al. 2018; Goldberg et al. 2020; reviewed by Svensson 2017), and abundant theory on evolution driven by within‐species interactions—resource competition (Ackermann and Doebeli 2004), cooperation (Axelrod and Hamilton 1981), or mate choice (Lande 1980)—and interactions with other species (e.g., predation, parasitism, etc.) (Abrams 2001; Senthilnathan and Gavrilets 2021). Such scenarios are prone to evolutionary feedbacks, because they cause natural selection to depend on the current genetic and phenotypic composition of the population; in other words, to be frequency dependent (Wright 1969), hereafter FD. In particular, negative frequency‐dependent selection (NFDS), where less common variants are favored (Wright and Dobzhansky 1946; Wright 1969), arises in ecological scenarios such as crypsis, where search images by predators penalize common prey types (Nosil et al. 2018): sexual conflict, where a similar process penalizes common female types via male harassment (Svensson et al. 2005; Rouzic et al. 2015), or self‐incompatibility in plants, where common pollen types have fewer pistils to fertilize (Wright 1939; Castric and Vekemans 2004). NFDS causes negative feedbacks, which often stabilize dynamical systems, but it may also yield cycling, or even complex dynamics (Lewontin 1958; Altenberg 1991; Gavrilets and Hastings 1995; Sinervo and Lively 1996).

Although most studies of adaptation tend to favor one or the other explanation (external forcing by the environment vs. internal feedbacks) for variation in natural selection, many real‐world situations likely include both. For instance, rising temperature may affect the way individuals within a species interact, through, for example, competition (Mitchell and Angilletta 2009; Germain et al. 2018) or mating (as recently shown for sex‐specific ornaments in dragonflies, Moore et al. 2021). More specifically, Svensson et al. (2020) recently showed that a female polymorphism maintained by negative frequency dependence was also under temperature‐dependent, frequency‐independent selection at an earlier life stage. Reciprocally, ecological interactions can modify the impacts of environmental change on organisms, such as ecological facilitation alleviating the detrimental effects of drought (Bruno et al. 2003). Therefore, a question that is likely highly relevant to many real‐life situations is: When a population is subject to both a changing external environment and internally driven dynamics caused by ecological interactions, which of these factors is likely to dominate the evolutionary dynamics? And how does the answer to this question influence the repeatability and predictability of selection and evolution?

These questions have received surprisingly little attention from evolutionary biologists. Svensson et al. (2005) simulated a combination of NFDS with environmental noise, and Svensson and Connallon (2019) recently investigated how FD affects adaptation and evolutionary rescue in a directionally changing environment. Rego‐Costa et al. (2018) showed that a cycling environment can modify the predictability of evolution for quantitative traits undergoing complex forms of FD that can lead to chaotic dynamics. Here, we ask more generally how FD affects the temporal variability and predictability of selection and evolution in a temporally fluctuating environment. Such a coupling between external forcing and internal feedbacks is an important element of realism for many populations in the wild, so our aim here is to provide a simple formalism to guide our understanding and prediction of their dynamics.

## When does frequency dependence alone cause predictable fluctuations?

Before proceeding further, it is worth clarifying when NFDS alone is likely to cause persistent fluctuations in selection. Throughout this work, we focus for simplicity on discrete polymorphisms determined by a single locus, as described in many empirical examples (Svensson 2017).

### INSIGHTS FROM A LOCAL STABILITY ANALYSIS

We first go back to a simple framework introduced by Lewontin (1958) to broadly characterize evolutionary dynamics generated by FD in discrete generations. Lewontin (1958) showed that alternative dynamic outcomes (stable equilibria, unstable equilibria, cycling) can be distinguished based on a simple metric, which we here denoted as *D*, defined as

(1) $$D={\left.\frac{\partial \Delta p}{\partial p}\right|}_{p=\hat{p}}.$$

**Figure 1:**
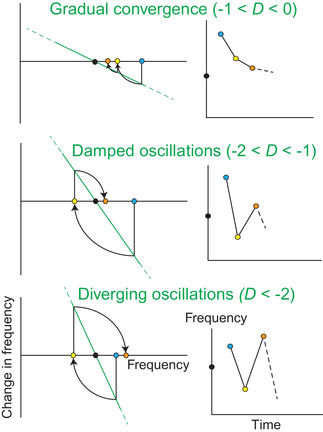
How the frequency dependence slope influences evolutionary dynamics. On the left panels, the green lines plot frequency change Δp against current frequency p. This relationship is approximated as linear near an equilibrium frequency $\hat{p}$ (black dot), and has negative slope under NFDS. Its steepness, measured by *D*, determines the frequency dynamics near $\hat{p}$. The cyan dot represents the initial frequency. Moving vertically toward the green line yields the corresponding frequency change, which increments the current frequency (via the circular arc arrow), yielding the frequency in the next generation (yellow dot). Iterating the process for one more generation yields orange dot. The system moves from gradual approach of equilibrium (top) to damped oscillations (middle) to diverging oscillations (bottom) as the steepness of the green line increases (larger negative *D*), as also illustrated by the frequency dynamics in the right panels.

In Figure 1, *D* is the slope of the green line plotting frequency change per generation Δp against frequency p, evaluated where it intersects the *x*‐axis (equilibrium frequency $\hat{p}$, black dot). Negative FD that may maintain polymorphism is characterized by a negative slope near an internal equilibrium (with $\hat{p}$ different from 0 or 1). Note that such relationship could also be explained by other forms of balancing selection (such as overdominance; see also *Discussion*), but we here assume it is caused by frequency‐dependent selection.

Indeed, starting at a small deviation from equilibrium $\delta \;=\;p-\hat{p}$, frequency change under selection can be approximated as linear in p, that is, Δp=Dδ. Iterating over multiple generations yields

(2) $$\delta_{t}={\left(1+D\right)}^{t}\;\delta_{0}.$$

Equation (2) makes it clear that D determines the system behavior near an equilibrium $\hat{p}$. When D>0 (positive FD), small initial deviations from equilibrium get amplified exponentially over time, and the equilibrium is unstable. In contrast, negative D leads to a diversity of outcomes. If −1<D<0, then $\delta_{t}$ decays exponentially over time, causing a gradual approach to the stable equilibrium $\hat{p}$ (Fig. 1, top), with timescale −1/ln(1+D) (faster with stronger NFDS, with *D* closer to −1). If −2<D<−1, the frequency overshoots its equilibrium in each generation, causing oscillations around $\hat{p}$ with period 2, alternating ups and downs. However, these oscillations are damped (Fig. 1, middle), and eventually vanish (persisting over a timescale of −1/ln[−(1+D)], longer when NFDS is stronger), and a stable equilibrium is again reached. Finally if D<−2, the frequency oscillates around the equilibrium but with exponentially increasing magnitude (Fig. 3, bottom).

This simple stability analysis (which applies more broadly to any discrete‐time dynamical system, e.g., Otto and Day 2007, pp. 163–170) shows that when Δp is well approximated as linear in p, the system moves from gradual approach to equilibrium, to damped oscillations, to unstable expanding oscillations, as the strength of NFDS increases (Fig. 1). Stable fluctuations, with fully predictable alternations of ups and downs of fixed magnitude around the equilibrium, only occur when D=−2 under this linear approximation, but transient fluctuations may still persist for some time if *D* is very close to −2 (for instance, the magnitude of fluctuations is halved in about 3, 7, and 13 generations if D=−1.8, −1.9, or −1.95, respectively).

### STRONG FREQUENCY DEPENDENCE CAN PRODUCE PREDICTABLE FLUCTUATIONS, BUT ALSO UNPREDICTABLE CHAOS

**Figure 2:**
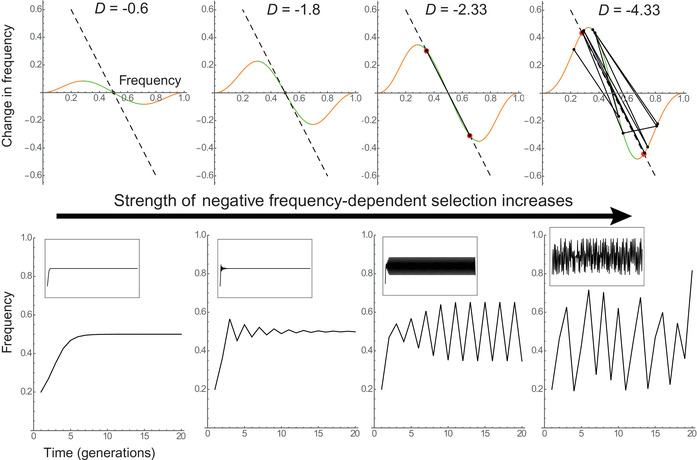
Fluctuations under nonlinear frequency dependence. Top row: The relationship between frequency change Δp and frequency p is shown for a diploid model of frequency dependence (adapted from Rice 2004). Green portions of the curve exhibit negative FD (downward slope), whereas orange portions have positive FD (upward slope). The slope *D* of the green portion at the point where it intersects the *x*‐axis increases from the left to the right panel. The dashed black line, with slope −2, may intersect the Δp curve at the red dots. Evolutionary trajectories over 10 generations (away from initial conditions) are shown as black lines and dots. They appear as single dots in the two leftmost panels because a stable equilibrium is reached, whereas in the third panel they overlap with the dashed black line in between the red dots. Bottom row: The frequency dynamics are represented over the first 20 generations (inset: 200 generations) for the same simulations. The sensitivity of heterozygote's fitness to their own frequency is *s*, whereas the sensitivity of each homozygote's fitness to the frequencies of the other genotypes is $s_{b}$ (more detail in Appendix S1). Parameter values are (from left to right): $s_{b}=\;1.5$ and s=0.75; $s_{b}=\;2.5$ and s=1.5; $s_{b}=\;3$ and s=1; $s_{b}=\;3$ and s=1.9.

We relied above on a local approximation near equilibrium, but the dependency of Δp on p cannot remain linear—or even just monotonic—over the full range of relative frequencies (from 0 to 1) if FD causes an internal equilibrium, because this would lead to unrealistically large frequency change near fixation. Indeed, frequency change under selection can generally be written as (Wright 1969; Crow and Kimura 1970)

(3) $$\Delta p=p\left(1-p\right)s\left(p\right),$$

where s(p) is a frequency‐dependent selection coefficient, and p(1−p) quantifies genetic diversity at the locus. As p(1−p)=0 when *p* = 0 or 1, frequency change Δp also must tend to 0 as alleles approach fixation. This implies that the simplest way for Δp to have a negative slope with respect to p near an internal equilibrium $\hat{p}$ is by having *positive* slopes at p=0 and p=1, as illustrated by orange portions of the curves in Figure 2. Hence, negative FD near an internal equilibrium may often imply positive FD near fixation (unless the fitness function is more complex), and the same holds for other forms of balancing selection such as overdominance (in fact, this was identified as a sufficient condition for protected polymorphism by Prout 1968). An important question therefore is: how likely is it that frequencies mostly remain within a region with negative slope, and near‐linear relationship, between Δp and p? As we show below, the answer largely depends on the slope *D* near equilibrium.

Previous work has shown that generalized diploid FD, where the fitness of all three genotypes at a biallelic locus (heterozygote and both homozygotes) depends linearly on all of their frequencies (Altenberg 1991; Gavrilets and Hastings 1995; Rice 2004; Cockerham et al. 2015), can lead to complex evolutionary dynamics, notably when heterozygotes exert strong detrimental effects on all genotypes, including themselves (as shown by Altenberg 1991; Gavrilets and Hastings 1995). We here rely on Rice's (2004) model (Appendix S1), focusing for simplicity on symmetric FD with an equilibrium frequency at $\hat{p}=\;1/2$ (as done by previous authors). The relationship between Δp and p can be highly nonlinear in this model (Fig. 2), but Lewontin (1958)’s simple criterion above still provides a useful guideline. When −2<D<0, the system behaves as predicted by its linear approximation near equilibrium: a stable equilibrium $\hat{p}\;$is reached regardless of initial frequency (Fig. 2, top left), preceded by damped oscillations if −2<D<−1 (Fig. 2, top middle‐left). In contrast for steeper slopes D<−2, the behavior is influenced by the nonlinearity of Δp with respect to p. In this symmetric model, the behavior of the system is then determined by where the line with slope −2 going through $\hat{p}$ (i.e., the 1−2p line, in dashed black in Fig. 2, top) intersects with the frequency change curve. If intersections occur in the part of the curve with negative FD (in green in Fig. 2), then a limit cycle is reached (independent of initial conditions), where frequencies oscillate between these intersections (red dots in Fig. 2). The magnitude of these fluctuations increases as the steepness of NFDS increases, causing the red dots in Figure 2 to move farther apart. Under very strong NFDS (D≪−2), FD is positive at the intersection (red dots in orange part of the curve, right panel of Fig. 2). The frequency p thus regularly explores regions with both positive and negative FD. Interestingly, this causes the dynamics to become chaotic, such that frequency change displays no obvious pattern, and slight differences in initial conditions can lead to very different evolutionary trajectories (Altenberg 1991; Gavrilets and Hastings 1995). When this occurs, even though the dynamics are completely deterministic, they cannot be predicted even over short timescales, because the strong dependency on initial conditions means that any measurement error is going to be amplified considerably.

In summary, NFDS by itself can only produce persistent frequency fluctuations if the relationship between Δp and p is very steep (D<−2). However, if this relationship is too steep, the system will regularly explore regions with positive and negative FD and become chaotic, so the fluctuations will not be predictable.

## How does frequency dependence affect the predictability of evolution in a fluctuating environment?

We have just seen that moderately strong NFDS (with −2<D<0) cannot maintain persistent fluctuations on its own. But what if an external perturbation, such as a temporally varying environment, interacts with the internal dynamics caused by FD?

### MODERATE NFDS CAN INCREASE THE PREDICTABILITY OF EVOLUTION IN UNPREDICTABLE ENVIRONMENTS

We start by considering perturbations that are themselves random, and thus unpredictable, such as environmental noise or genetic drift. For simplicity, we rely on Lewontin's (1958) linearized model of NFDS (Fig. 1), and add a noise component to it. When the equilibrium is attracting (−2<D<0), the recursion for $\delta \;=\;p-\hat{p}$ becomes

(4) $$\delta_{t+1}=\left(1+D\right)\;\delta_{t}+\sigma W_{t},$$

where $W_{t}$ is drawn from a standard normal (white noise) and σ is the standard deviation of the noise process. Beyond the assumption of linear FD, equation (4) further assumes that noise variance of frequency change is independent of frequency, which is generally not true under fluctuating selection (Wright 1948; Kimura 1954; Gillespie 1973; Chevin 2019), but may be a good approximations if frequencies remain sufficiently close to 1/2.

Equation (4) implies that in a white noise (non‐autocorrelated) environment, δ may be approximated as a first‐order autoregressive process (AR1). Such a process is stationary, such that the variance of random fluctuations in frequency eventually reaches a constant value, $V\;\left(\delta \right)=\;V\;\left(p\right)=\;-\frac{\sigma^{2}}{D(2+D)}$, which is highest toward D=−2 and D=0, and minimum at D=−1, where it equals the variance of the external perturbation, $V\;\left(p\right)=\sigma^{2}$. In finite populations, $\sigma^{2}$ also includes a component caused by genetic drift, with variance $\frac{p(1-p)}{2N_{e}}$ (in diploids), but this component should cause moderate fluctuations unless the variance effective population size $N_{e}$ is very small.

In the presence of noise, although fluctuations in frequency are random, they still have some predictable aspects. In particular, frequency change, which is often the main focus in studies of evolutionary dynamics (Nosil et al. 2018; Goldberg et al. 2020), has autocorrelation $\rho \;\left(\Delta p\right)=\frac{D}{2}$ over one generation. Hence, frequency changes are negatively autocorrelated under NFDS, all the more so as the absolute strength of FD increases, but with no influence of the magnitude of noise, as long as noise exists and can be accurately modeled by equation (4). For large negative slopes (D→−2), autocorrelation tends toward ρ(Δp)=−1, such that increases in frequency are almost certainly followed by decreases in frequency of similar magnitude (and vice versa). The short‐term predictability of evolution can be defined as the proportion of variance in frequency change that is explained by the previous frequency change. From equation (4), this is simply

(5) $$\rho^{2}\left(\Delta p\right)=\frac{D^{2}}{4},$$

which saturates at its maximum of 1 for D=−2, and should remain high even under stronger FD (D<−2), as long as the dynamics are not chaotic (see below). This analysis can easily be extended to the case where noise is itself autocorrelated. For instance if the noise process is autoregressive of order r, then fluctuations in allelic frequency become autoregressive of order r+1 (Karlin and Taylor 1981; Box et al. 2008). These have more complex dynamics, with r+1 embedded timescales providing more “memory” to the process, but they should still be characterized by rapid fluctuations around the equilibrium frequency $\hat{p}$ as long as −2<D<−1.

To investigate the robustness of these predictions to the approximations in equation (4), we carried out simulations with randomly fluctuating selection. Without FD, maintenance of polymorphism in such temporally varying environments is possible when temporal variation in selection leads to associative overdominance, whereby the long‐term, geometric mean fitness of the heterozygote is larger than those of both homozygotes (marginal overdominance), even when there is no overdominance in any specific generation (Haldane and Jayakar 1963). This may occur under beneficial reversal of dominance, such that that the heterozygote's fitness is always closer to that of the most‐fit homozygote (Posavi et al. 2014; Wittmann et al. 2017), or even without dominance in any generation (Haldane and Jayakar 1963; Lande 2008). Here, we model the latter for simplicity, by assuming that selection on codominant alleles is reversed symmetrically across environments (following Lande 2008), and combine this with the diploid FD model in Figure 2 (more details in Appendix S1).

**Figure 3:**
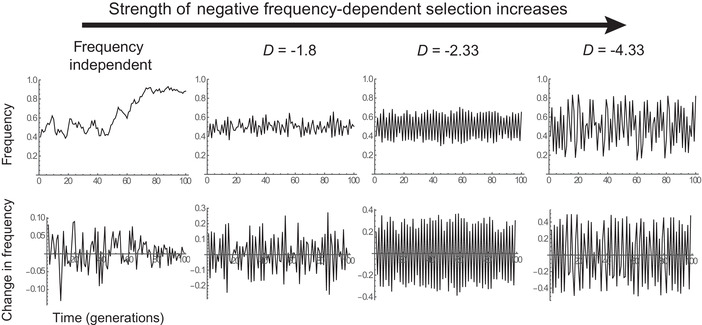
Frequency dependence in a randomly fluctuating environment. The dynamics of allelic frequency (upper row) and frequency change (lower row) are shown in a diploid model of fluctuating selection caused by a random environment, combined with diploid frequency dependence as in Figure 2. The fluctuating environment causes the selection coefficient of homozygotes to fluctuate randomly over time, with mean 0 and standard deviation 0.2. The frequency‐dependent part of the model is as in Figure 2, with parameter values (from left to right panel):$\;s_{b}=\;0$ and s=0 (no frequency dependence); $s_{b}=\;2.5$ and s=1.5; $s_{b}=\;3$ and s=1; $s_{b}=\;3$ and s=1.9.

Figure 3 shows that without FD, the frequency fluctuates erratically under the influence of the random environment, with a temporal mean of *p* = 1/2 set by marginal overdominance. Under intermediate NFDS (D=−1.8), frequency fluctuates less erratically than without FD, instead displaying alternations of ups and downs around the equilibrium frequency $\hat{p}=\frac{1}{2}\;$. Remarkably, this FD strength would lead to damped oscillations in a constant environment (Fig. 2), but when combined with a random environment these oscillations are maintained indefinitely as quasi‐cycles, through a phenomenon called stochastic resonance (Nisbet and Gurney 1976; Boettiger 2018). Under stronger FD (D=−3), fluctuations display internally driven two‐generation cycles, with a magnitude influenced by the random perturbations, but not much their general pattern. Lastly under very strong FD, fluctuations become erratic again, and with much larger magnitude than those caused by the randomly fluctuating environment, as a result of chaos driven by FD.

**Figure 4:**
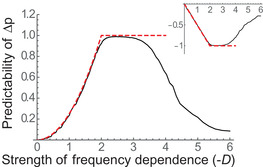
Predictability of evolution with frequency dependence in a randomly changing environment. The predictability of evolution, as measured by the squared autocorrelation of frequency change over one time step, is represented against the strength of NFDS, for a population that undergoes randomly fluctuating selection combined with diploid frequency dependence. The red dashed line shows the analytical expectation under linear frequency dependence (eq. 5) for 0≤−D≤2, followed by saturation at 1 for −D>2. The true predictability closely matches this prediction up to −D≈3, beyond which chaotic dynamics reduce it. The inset shows the autocorrelation, with negative expectation D/2 for 0≤−D≤2. A single simulation was run for 5000 generations, of which the first 200 were removed to compute the autocorrelation. The parameters for the randomly fluctuating environment are the same as in Figure 3. For the frequency dependence, we used the same model as in previous figures, with $s_{b}=\;0$ to 3 and s=0 for all 0≤−D≤1.8, and with $s_{b}=\;3$ and s=0 to 2.1 for all 1.8≤−D≤6.

Figure 4 shows how the temporal predictability of frequency change over one generation depends on the strength of frequency dependence in these simulations. Predictability is 0 without FD, as expected because environmental forcing is white noise. Our simple approximation that assumes linear frequency dependence (eq. 5, dashed red line in Fig. 4) works remarkably well over the entire range over which it is defined (0≤−D≤2), and even though the true FD of Δp is clearly not linear (Fig. 4). The autocorrelation of these fluctuations is negative (inset in Fig. 4), because NFDS causes alternations of ups and downs. The predictability of fluctuations remains close to its maximum of 1 under stronger frequency dependence 2≤−D≤3 (horizontal red dashed line in Fig. 4), because FD then causes predictable internal fluctuations, which are only marginally perturbed by the random noise (as seen in Fig. 3). Beyond this point, FD starts to decrease the predictability of evolution, because the dynamics become chaotic. Interestingly, the transition in predictability is not abrupt as chaos arises. This is perhaps because the contribution of chaos to predictability depends on how the magnitude of chaotic fluctuations relates to that of random noise in selection.

### STRONG NFDS CAN DECREASE THE PREDICTABILITY OF EVOLUTION IN A PREDICTABLE ENVIRONMENT

Let us now turn to the opposite situation, where the changing environment is highly predictable, but FD perturbs evolutionary dynamics in a way that reduces their predictability. We illustrate this scenario by considering a highly predictable aspect of seasonality (e.g. photoperiod), causing yearly cycles in selection with a period of 20 generations, consistent with observed seasonal fluctuations of allelic frequencies across the genome of *Drosophila* in North America (Bergland et al. 2014; Wittmann et al. 2017). We use the same model as above for the influence of the environment on selection (Haldane and Jayakar 1963; Lande 2008), but now assume that this environment is periodic, such that the selection coefficient has mean 0, and cycles from positive to negative once every year, which lasts 20 generations (Appendix S1).

**Figure 5:**
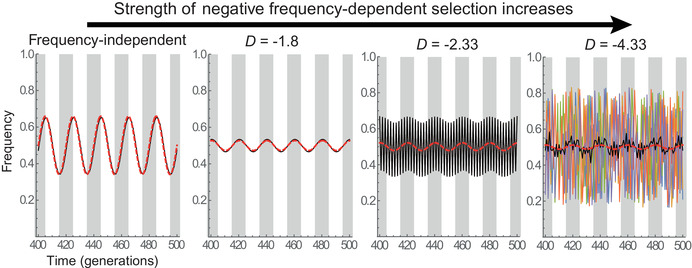
Frequency dependence in a periodic environment. The dynamics of allelic frequency are represented for simulations starting from slightly different initial conditions at time 0 (SD of initial frequency:$\;10^{-3}$). The black line shows the average over 100 replicates, and five individual replicates are also represented as colored lines (only visible in the rightmost panel). The red dashed lines show analytical predictions without (left) or with frequency dependence. The fluctuating environment causes the frequency‐independent selection coefficient of homozygotes to undergo a deterministic sine wave (amplitude 0.2, period 20), materialized by the gray shadings when selection coefficients are positive. Generations 400–500 are represented to ensure that the stable cycles are reached where relevant, but the chaotic dynamics on the right appear in the first few generations, as in Figure 2. The FD part of the model is as in Figure 2, with parameter values (from left to right panel):$\;\;s_{b}=\;0$ and s=0 (no frequency dependence); $s_{b}=\;2.5$ and s=1.5; $s_{b}=\;3$ and s=1; $s_{b}=\;3$ and s=1.9.

The strength of FD also has large impacts on evolutionary dynamics in this context. Without FD, allelic frequencies settle into periodic fluctuations, well approximated by a sine wave with the same period *T* as the selection coefficient. The amplitude of these cycles is approximately multiplied by $\frac{T}{8\pi }$ relative to cycles in selection (Appendix S1). This amplitude increases with increasing period of fluctuations, because more generations per year allow more accumulation of frequency change in each cycle. In addition, cycles in frequency lag behind cycles in selection by a quarter period (as shown in Appendix S1), such that increases in frequency coincide with positive selection coefficients (gray shading in Fig. 5). These analytical predictions appear as red dashed line in Figure 5, left.

NFDS modifies these patterns in a number of ways. When the strength of FD is appreciable, but not sufficient to cause fluctuations by itself, the cycles retain the same period as the environment, but with a smaller amplitude, and a shifted phase. Approximating FD as linear as previously (Appendix S1), the amplitude of fluctuations is multiplied by

(6) $$R_{A}=\frac{2\pi }{\sqrt{4\pi^{2}+D^{2}T^{2}}},$$

relative to the case without FD (Appendix S1). $R_{A}$ is at most 1 under weak FD and rapid environmental fluctuations ($D^{2}T^{2}\ll 4\pi^{2})$, and decreases with increasing absolute strength of FD and period of environmental fluctuations, tending toward $\frac{2\pi }{DT}$ when both are large. The periodic lag, or phase shift, between the dynamics of allelic frequencies and fluctuating selection is approximately

(7) $$L=\frac{\mathrm{ArcTan}\left[-\frac{2\pi }{DT}\right]}{2\pi }\;,$$

which tends to 1/4 (as without FD) under weak FD (−DT≪2π), but decreases with increasing strength of FD, tending to 0 under strong FD (−DT≫2π). Hence, as the strength of FD increases, the frequency cycles are increasingly buffered, and synchronized with cycles in selection, such that the highest frequency coincides with the largest selection coefficient (Fig. 5, second panel; gray shadings are periods with positive selection coefficients).

Under stronger NFDS (D=−2.3 in Fig. 5), FD by itself generates cycles with period 2, superimposed on the buffered cycles of period 20 caused by the fluctuating environment (which are still well described by the analytical prediction, dashed red line in Fig. 5). Although the pattern of fluctuations is more complex, evolutionary trajectories remain fully repeatable (all replicates are confounded with their average, black line in Fig. 5). This is in sharp contrast with what happens under very strong FD (D=−4.33 in Fig. 5). In this chaotic regime, even minute differences in initial conditions lead to completely different and erratic evolutionary trajectories (colored lines for different replicates), such that the average trajectory (in black) displays no clear pattern over time, and tends toward the equilibrium frequency $\hat{p}=\frac{1}{2}\;$.

**Figure 6:**
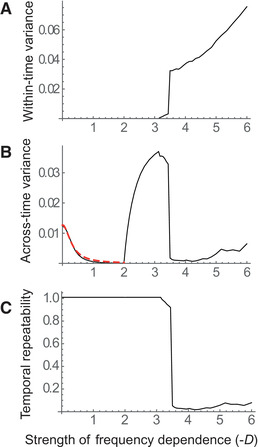
Variability and repeatability of evolution with frequency dependence in a predictable environment. For each strength of frequency dependence, 500 replicate simulations were run starting from slightly different initial conditions (SD of initial frequency:$\;10^{-3}$), and the variability of evolutionary trajectories (allelic frequencies over time) was computed over the last 200 out of 300 generations. Panel A shows the variance among replicates at each time point, averaged over time (within‐time variance). Panel B shows the temporal variance in the mean trajectory (across‐time variance), with the red dashed line representing the analytical approximation assuming linear frequency dependence. Panel C shows the repeatability of trajectories, measured as the proportion of the total variability explained by the mean evolutionary trajectory over time. Repeatability equals 1 when all replicates perfectly track the mean trajectory, and tends to 0 when replicate trajectories fluctuate independently from each other. The parameters for the periodic environment are the same as in Figure 5. For the frequency dependence, we used the same model as in previous figures, with $s_{b}=\;0$ to 3 and s=0 for all 0≤−D≤1.8, and with $s_{b}=\;3$ and s=0 to 2.1 for all 1.8≤−D≤6.

Figure 6 shows how the variability and repeatability of evolutionary trajectories changes with the strength of FD in such a periodic environment. A sharp threshold can be seen toward D=−3.5. Below this threshold, there is essentially no variance across replicates starting from very similar initial conditions (Fig. 6A), as they all converge to limit cycles determined by the environment (and possibly also by FD). Above the threshold, the variance among replicates first increases abruptly, then keeps increasing more smoothly with the strength of FD. The temporal variance of evolutionary trajectories (Fig. 6B), which corresponds to the variance over time of the black line in Figure 5, first decreases with increasing strength of FD, consistent with the buffering effect of FD (eq. 6). This decline over 0≤−D≤2 is well captured by our analytical approximation (red dashed line in Fig. 6B). However, when FD becomes strong enough to cause fluctuations by itself (2≤−D≤3.5), the temporal variance increases with increasing FD, because steeper FD should lead to fluctuations of higher magnitude (Fig. 2). Finally under chaotic fluctuations (−D≥3.5), the absence of consistent pattern across replicates translates into a mean trajectory that does not vary much over time.

These effects can be summarized by computing the repeatability of evolutionary trajectories, defined as the proportion of their total variance that is explained by the variance in the mean trajectory over time (as used by Rego‐Costa et al. 2018 in a similar context). This measures the extent to which evolution in one replicate can be predicted from the average of other replicates. Strikingly, the repeatability of evolution in a periodic environment remains close to 1 for all FD strengths that do not produce chaos (−D≤3.5), but then suddenly shifts to almost 0 past this threshold. Hence, evolution in response to a predictable environment can switch abruptly from highly predictable to highly unpredictable as the strength of FD increases, causing the dynamics to become chaotic.

## Discussion

The combination of internal feedbacks caused by ecological interactions with external forcing caused by a changing environment is likely to be common and widespread in nature (Germain et al. 2018; Svensson and Connallon 2019; Svensson et al. 2020; Grainger et al. 2021; Moore et al. 2021). We thus wished to understand (1) how frequency dependence interacts with a changing environment (or equivalently, random perturbations caused by genetic drift) in driving evolutionary dynamics, and (2) how this impacts the pattern and predictability of evolution. Our analysis reveals that whether, how, and how strongly FD influences evolutionary dynamics and their predictability crucially depends on the strength of FD and on how FD interacts with a changing environment. In addition, we show that a simple criterion proposed over 60 years ago by Lewontin (1958) serves as a very useful yardstick for understanding these dynamics, even in regimes it was not originally designed for.

In the absence of any external perturbation, FD of moderate strength is unlikely to maintain predictable patterns of frequency change for long in large populations. Fluctuations are instead likely to be transient, leading to a stable equilibrium. Although a stable equilibrium is predictable in a sense, and absence of evolution can inform about the existence of selective processes (Eldredge et al. 2005), a constant frequency would generally not be analyzed in terms of the predictability of evolutionary dynamics. Very strong FD can maintain long‐term, predictable fluctuations in frequencies, but may also lead to unpredictable chaotic dynamics (Altenberg 1991; Gavrilets and Hastings 1995).

Although this may suggest the FD should not influence patterns of fluctuating selection unless it is very strong, this is not necessarily true. The reason is that (i) virtually any population is exposed to temporal changes in its natural environment, causing natural selection to vary over time (Reimchen 1995; Grant and Grant 2002; Bell 2010; Chevin et al. 2015; de Villemereuil et al. 2020), and (ii) FD can alter evolutionary responses to such temporally varying selection. Interestingly, we here show that even FD that would be too weak to maintain long‐term fluctuations by itself can still induce partly predictable fluctuations, when noise also perturbs frequency change. Here, we assumed that this noise was caused by a randomly fluctuating environment, but it may also be due to genetic drift, with the relative importance of these two sources of randomness depending on the product of the variance in selection by the effective population size (Chevin 2019). Drift may thus be likely to play a more prominent role in vertebrates (e.g, side‐blotched lizards, Sinervo and Lively 1996; Sinervo et al. 2000) than it does in insects (Oxford 2005; Rouzic et al. 2015). Regardless of its origin, when noise is added to an evolutionary system subject to FD, it can reveal its intrinsically cycling nature. This occurs because noise causes the system to enter a regime known as stochastic resonance (Nisbet and Gurney 1976; Boettiger 2018), where it undergoes quasi‐cycles that are much more predictable than the noise itself (Figs. 3 and 4). An unexpected consequence of this phenomenon is that factors thought to decrease the predictability of evolution (unpredictable environmental noise, or drift) can actually contribute to establishing persistent, partly predictable fluctuations in frequency. Noise can therefore contribute to improving information about evolutionary processes, as previously described for ecology (Boettiger 2018). Or to put it differently, NFDS can transform inherently unpredictable evolutionary responses to stochastic noise into largely predictable ones. Previous work had suggested that random perturbations may be necessary to reveal the fluctuations inherent to NFDS (Svensson et al. 2005; Rouzic et al. 2015), but we here demonstrate this principle more formally, and quantify it. For instance, our results in Figure 4 confirm the intuition by Oxford (2005) that an almost flat relationship between Δp and p where it crosses the *x*‐axis (*D* close to 0) would lead to little contribution of NFDS to frequency change and weak predictability of evolution over most observed range of frequencies.

At the other end of the spectrum, FD can interfere with highly predictable dynamics driven by a periodic environment, such as seasonality. First, FD that is too weak to lead to fluctuations on its own can still buffer evolutionary responses to periodic cycles in the environment, as illustrated in Figures 5 and 6. This buffering may make the influence of the periodic environment more difficult to detect empirically. Stronger FD further causes cycles with their own periodicity, which may conceal the influence of the periodic environment (in addition to also buffering it). Finally, extremely strong FD can lead to chaotic dynamics, making evolution highly unpredictable because of a strong dependence on initial conditions, thus overwhelming the responses to the predictable environment.

Strikingly, this diversity of outcomes is well predicted by a simple criterion proposed by Lewontin (1958), based on the slope *D* of frequency change against frequency near an equilibrium frequency (eq. 1 and Fig. 1). This criterion, typical of stability analysis (Otto and Day 2007), was designed for constant environments, but also largely drives evolutionary outcomes when FD is combined with a changing environment (Figs. 3, 4, 5, 6), so it should be a key ingredient for understanding and predicting evolution in this context. Empirical estimates for *D* can be extracted from a few examples from the literature. Goldberg et al. (2020) recently reported a slope of D=−0.23 for changes in the frequencies of morphs of the plant *Datura wrightii* over 20 years. Nosil et al. (2018) studied changes in the frequency of a striped morph among all green morphs of the walking stick *Timema cristinae* over 18 years; by reanalyzing their dataset, we find that D=−1.06. Similarly reanalyzing the dataset of le Rouzic et al. (2015), which consists of multiple populations of the damselfly *Ischnura elegans*, we find D=−0.95 for the frequency of a male mimic morph in females. Wright and Dobzhansky (1946) analyzed changes in the frequencies of inversions in experimental populations of the fruit fly *Drosophila pseudoobscura*, over three to four generations in the laboratory. Transforming from their slightly different estimate of frequency dependence (Appendix S1), we find D=−0.27. In all these examples, the strength of FD is thus moderate, but not weak: it falls within the interesting range where NFDS would not cause persistent fluctuations on its own, but can modify responses to a fluctuating environment (Figs. 3, 4, 5, 6). This is all the more striking as the initial aim of Wright and Dobzhansky's (1946) experiment was to reproduce experimentally, and thus better understand, seasonal cycles in frequency, as still currently observed in fruit flies using genomic data (Bergland et al. 2014). Similarly, the demonstration by Svensson et al. (2020) that temperature drives a frequency‐independent component of viability selection on female color morphs in *I. elegans* damselflies suggests that seasonality could lead to periodic selection in this species (although at a within‐generation timescale).

That the parameter *D* captures important features of evolutionary dynamics with FD does not mean that it is sufficient by itself to understand how selection operates in any particular system. Indeed, *D* is a very summarized metric, and different selective scenarios may lead to undistinguishable slopes, or even overall relationships, between Δp and p. This was already emphasized by Wright and Dobzhansky (1946), who showed that the relationship between Δp and p that they observed was as consistent with frequency dependence as it was with (possibly sex‐specific) overdominance. A formal demonstration of FD thus requires demonstrating that the individual (or marginal) fitness of each genotype/phenotype depends on the genetic/phenotypic composition of the population, as done experimentally in, for example, guppies (Olendorf et al. 2006), sticklebacks (Bolnick and Stutz 2017), stick insects (Nosil et al. 2018), or *Heliconius* butterflies (Chouteau et al. 2016, involving positive rather than negative FD). On the other hand, FD of *individual fitness* only leads to FD *selection* if genotypes/phenotypes differ in how their fitness depends on frequency.

We have used one of the simplest population genetic models of FD at a single biallelic locus (leading to, e.g., discrete morphs), allowing the argument to be expressed in terms of empirically accessible quantities. This is in line with most empirical investigations of FD in the wild, which have typically focused for simplicity on discrete categories, such as color polymorphisms (Sinervo and Lively 1996; Halkka et al. 2001; Oxford 2005; reviewed by Svensson 2017). Nevertheless, the prevalence of discrete traits in work on FD is only witness to their ease of study, and many ecologically relevant traits instead exhibit polygenic, quantitative heritable variation (Walsh and Lynch 2018). There is no reason why FD selection should be less prevalent for quantitative traits, although it is clearly less investigated. FD selection can readily be inferred empirically on quantitative traits, by including phenotypes of interactors when estimating fitness surfaces (Wolf et al. 1999; Santostefano et al. 2020). On the theoretical side, FD selection on quantitative traits has long been modeled, by letting the individual fitness function depend on the mean phenotype, or other aspects of the phenotype distribution (Slatkin 1979; Doebeli 1996; Burger and Gimelfarb 2004; Svensson and Connallon 2019). However, understanding whether a simple metric (such as *D*) also delineates evolutionary outcomes in this context—including in a changing environment—would require further work. For instance, evolutionary theory has made it clear that typical measurements of selection on quantitative traits (selection gradients and differentials) need to be handled with care in the presence of FD (Lande 1976; Abrams et al. 1993). In addition, some evolutionary outcomes may differ qualitatively for quantitative traits. For instance, the evolutionary dynamics of quantitative traits may remain partly predictable even when chaotic, if environmental fluctuations are larger than the chaotic attractor, such that the mean phenotype still overall tracks a periodic optimum phenotype (Rego‐Costa et al. 2018), whereas frequencies of discrete morphs are necessarily bounded between 0 and 1.

Even with discrete types, evolutionary dynamics under NFDS could differ from our model in a number of ways. First, we assumed discrete nonoverlapping generations, which are generally more prone to fluctuations in ecology and evolution (e.g., May 1976). Interestingly, most of the empirical examples highlighted above (from univoltine insects to short‐lived lizards) are in fact very close to having discrete nonoverlapping generations, which may explain why they also display fluctuations in the field. Second, eco‐evolutionary feedbacks may be more complex than can be summarized by a simple dependence of selection on frequency. For instance, such feedbacks may materialize as a combination of FD with density dependence, mediated by environmental factors such as resources or interacting species (Heino et al. 1998; Lion 2018). Interestingly, such an interplay of FD selection with density‐dependent *r*/*K*‐selection was shown to cause persistent fluctuations in the frequencies of female color morphs in the side‐blotched lizard (Sinervo et al. 2000), whereas intransitive rock‐paper‐scissor FD interactions with no density dependence only led to transient fluctuations for male color morphs in the same species (Sinervo and Lively 1996; Sinervo 2001). Likewise, an interplay between FD and density‐dependent selection (along with selection based on temperature) appears to act in *Timema* stick‐insects (Farkas and Montejo‐Kovacevich 2014; Nosil et al. 2018). Third, when the changing environment is an interacting species (predator, competitor, parasite, etc.), internal dynamics may influence the external forcing, causing temporal changes in the FD function illustrated in Figures 1 and 2. All these scenarios would be worth investigating thoroughly in future studies.

Despite these complexities and challenges, our simple theoretical results may help understand and interpret temporal microevolutionary patterns, by providing clear predictions based on population metrics that are relatively simple to obtain empirically (e.g., Wright and Dobzhansky 1946; Rouzic et al. 2015; Nosil et al. 2018; Goldberg et al. 2020), ideally coupled with manipulative, individual‐level evidence (Olendorf et al. 2006; Chouteau et al. 2016; Bolnick and Stutz 2017). Our hope is that this work will stimulate empirical approaches that account for what should be an important aspect of many evolutionary systems: an interplay of internal dynamics caused by frequency‐dependent interactions, with external forcing caused by a changing environment.

## AUTHOR CONTRIBUTIONS

LMC, ZG and PN conceived the study. LMC designed and analyzed the models, and wrote the initial version of the manuscript. ZG and PN contributed to later versions of the manucript.

## CONFLICT OF INTEREST

The authors declare no conflict of interest.

## Supporting information

Online Appendix to Chevin, Gompert, & Nosil: Frequency dependence and the predictability of evolution in a changing environment.

Supplementary information
