# Supplementary material for: Frequency dependence and the predictability of evolution in a changing environment
Source: Evol Lett. 2021 Dec 20;6(1):21–33. doi: 10.1002/evl3.266 (PMC8802243; doi:10.1002/evl3.266)
Supplement: Supplementary file 1 — Online Appendix to Chevin, Gompert, & Nosil: Frequency dependence and the predictability of evolution in a changing environment. [file EVL3-6-21-s001.pdf]

Online Appendix to Chevin, Gompert & Nosil:
Frequency dependence and the predictability of evolution in a changing environment.
Published in Evolution Letters.

Details of the models

Diploid frequency dependence

To investigate cycles or chaos caused by frequency-dependent selection, we relied on a model by Rice (2004), itself modified from previous models of frequency dependence in diploids (Altenberg 1991; Gavrilets and Hastings 1995; Cockerham et al. 2015), by letting selection coefficients (instead of fitnesses) be frequency dependent. The model focuses on a bi-allelic locus, where the relative fitnesses w_{i} of the three diploid genotypes AA, Aa and aa are linear combinations of their frequencies f_{i},

$$\begin{pmatrix} w_{\mathrm{AA}} \\ w_{\mathrm{Aa}} \\ w_{\mathrm{Aa}} \end{pmatrix} = \begin{pmatrix} 1 \\ 1 \\ 1 \end{pmatrix} + \mathbf{W}. \begin{pmatrix} f_{\mathrm{AA}} \\ f_{\mathrm{Aa}} \\ f_{\mathrm{aa}} \end{pmatrix}. \tag{A1}$$

The matrix **W** describes the sensitivity of each genotype's fitness to the frequencies of all genotypes (including itself, along the diagonal). It emerges from how interactions between individuals depend on their genotypes and phenotypes. Previous work has made it clear that cycle and chaos can occur when the heterozygote has detrimental effects on all genotypes (Altenberg 1991; Gavrilets and Hastings 1995; Rice 2004). For simplicity we retain the symmetry assumptions from these previous studies (whereby the homozygotes are interchangeable in terms of fitness), and further simplify the model following Rice (2004) by only keeping two parameters: s for the dependence of heterozygote fitness on their own frequency, and s_b for the frequency dependence in homozygotes (s_b was fixed to 3 in Rice 2004). We thus have

$$\mathbf{W} = \begin{pmatrix} 0 & -s_b & s_b \\ 0 & -s & 0 \\ s_b & -s_b & 0 \end{pmatrix}, \tag{A2}$$

such that the fitness of homozygotes not only decreases with increasing frequency of heterozygotes, but also increases with increasing frequency of the other homozygote, which also leads to a form of negative FD. From these frequency-dependent fitnesses, we obtained the genotype frequencies after selection classically as $f_i' = f_{\mathrm{i}} w_{\mathrm{i}} / \bar{w}$ (where $\bar{w} = \sum_i f_{\mathrm{i}} w_{\mathrm{i}}$ is the mean fitness), and frequencies in the next generations by assuming Hardy-Weinberg equilibrium under random mating. In this model, it can be shown that the slope of frequency dependence defined eq. (1) is

$$D = \frac{3s_b - 2s}{s_b + 2s - 8} \quad \text{(A3)}$$

Environmental change in selection

To model a variable environment causing persistent fluctuations in allelic frequencies, we used a model modified from Haldane and Jayakar (1963), where temporal change in the relative fitnesses of homozygotes leads to associative overdominance. More precisely, we assumed that the relative fitnesses of genotypes AA, Aa and aa are $1 + s_F(t)$, 1, and $1 - s_F(t)$, where $s_F(t)$ is a temporally changing selection coefficient responding to a changing environment. If $s_F(t)$ varies over time without bias (ie, if it averages to 0 over time), then polymorphism is maintained over time due to associative overdominance, but allelic frequencies may still fluctuate in response to fluctuating selection.

To model a random environment, we assumed white noise for simplicity, and drew $s_F(t)$ from a normal distribution with mean 0 and variance σ_F^2. To model predictable, cyclical (eg seasonal) change in selection, we use a cosine function with amplitude A (the maximum selection coefficient) and period T,

$$s_F(t) = A\cos\left(\frac{2\pi t}{T}\right). \quad \text{(A4)}$$

Finally, when combined with frequency-dependent selection, assumed to corresponds to an independent episode of viability selection, the overall fitnesses for the three diploid genotypes are

$$\begin{pmatrix} w_{\text{AA}} \\ w_{\text{Aa}} \\ w_{\text{Aa}} \end{pmatrix} = \begin{pmatrix} 1 + s_F(t) & 0 & 0 \\ 0 & 1 & 0 \\ 0 & 0 & 1 - s_F(t) \end{pmatrix} \left[\begin{pmatrix} 1 \\ 1 \\ 1 \end{pmatrix} + \begin{pmatrix} 0 & -s_b & s_b \\ 0 & -s & 0 \\ s_b & -s_b & 0 \end{pmatrix} \begin{pmatrix} f_{\text{AA}} \\ f_{\text{Aa}} \\ f_{\text{aa}} \end{pmatrix} \right]. \quad \text{(A5)}$$

Analysis in a periodic environment

In the periodic regime defined by eq. (A4), allelic frequencies eventually settle into cycles around their average of $p = \frac{1}{2}$. Without frequency dependence, combining the periodic selection coefficient above with the classic recursion for frequency change (eq. (3) in the main text), and approximating the dynamics in continuous time, leads to the differential equation

$$\frac{dp}{dt} = p(1-p)A\cos\left(\frac{2\pi t}{T}\right) \approx \frac{A}{4}\cos\left(\frac{2\pi t}{T}\right), \quad \text{(A6)}$$

where the rightmost member in eq. (A6) comes from assuming that selection is weak enough that frequencies stay close to their average ½, such that $p(1-p) \approx \frac{1}{4}$. Solving for p leads to

$$p \approx \frac{1}{2} + \frac{AT}{8\pi}\cos\left[2\pi\left(\frac{t}{T} - \frac{1}{4}\right)\right]. \quad \text{(A7)}$$

Equation (A7) indicates that allelic frequencies approximately follow sinusoidal cycles with the same period as the selection coefficient, but lagging by a quarter of a cycle, and with amplitude multiplied by $\frac{T}{8\pi}$. The exact solution to eq. (A6) (not assuming that $p(1-p) \approx \frac{1}{4}$) is $p = \frac{1}{1+\frac{1-p_0}{p_0}\text{Exp}\left\{-\frac{AT}{2\pi}\cos\left[2\pi\left(\frac{t}{T}-\frac{1}{4}\right)\right]\right\}}$, *i.e.* a logistic function of a sine wave with same period and phase as in eq. (A7). This will be well approximated by the sine wave in eq. (A7) as long as the amplitude is not large (AT small), such that frequencies do not expand much above and below 0.5. For larger AT, the exact solution is bounded to

remain between 0 and 1, while the approximation in eq. (A7) is not.

If we also include frequency dependence, approximated as linear and with slope D near the equilibrium at $\hat{p} = 1/2$, then eq. (A6) becomes (still assuming that $p(1-p) \approx \frac{1}{4}$)

$$\frac{dp}{dt} \approx \frac{A}{4} \cos\left(\frac{2\pi t}{T}\right) + D(p - 1/2). \tag{A8}$$

The asymptotic solution for the dynamics of frequencies, after the influence of initial conditions has vanished (in non-chaotic regimes), is after some algebra,

$$p \approx \frac{1}{2} + \frac{AT}{4\sqrt{4\pi^2 + D^2T^2}} \cos\left[2\pi\left(\frac{t}{T} - L\right)\right]. \tag{A9}$$

The temporal variance of these cycles, obtained by averaging $(p - \frac{1}{2})^2$ over a period, is simply half the squared amplitude, leading to

$$V(p|cycle) = \frac{A^2T^2}{32(4\pi^2 + D^2T^2)}. \tag{A10}$$

The periodic lag, or phase shift, between the dynamics of allelic frequencies and that of selection is

$$L = \frac{\text{ArcTan}[-\frac{2\pi}{DT}]}{2\pi}. \tag{A11}$$

From the properties of the arctangent function, under weak FD ($-DT \ll 2\pi$) the lag L tends towards 1/4 of a cycle, as without FD. Under strong frequency dependence ($-DT \gg 2\pi$), L tends to 0, such that the cycles in allelic frequencies are synchronized with those of selection coefficients. The overall behavior of the lag behind the selection coefficient is represented below as a function of the strength of FD, for different periods of the cycling environment (5, 10, and 20, as curves become darker).

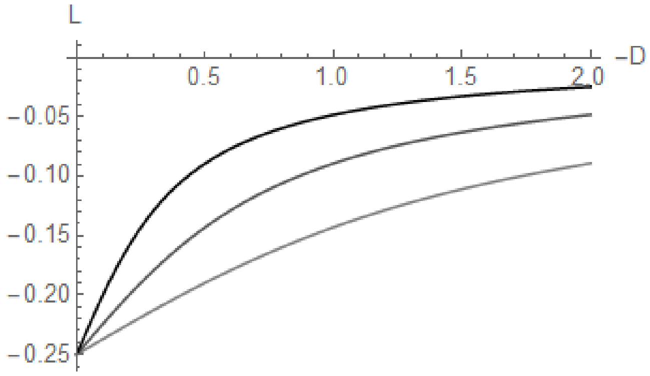

Empirical estimates of the strength of frequency dependence.

Wright and Dobzhansky (1946) estimated two parameters a and b, such that (from their eqs. 23-24)

$$\Delta p = p(1-p)\frac{a - bp}{1 - (a - bp)(1 - 2p)}. \tag{A12}$$

The equilibrium occurs for $\hat{p} = b/a$. Taking the derivative of Δp with respect to p at $\hat{p}$ then yields

$$D = \frac{a(a-b)}{b}, \tag{A13}$$

which combined with the reported estimates for a = .902 and b = 1.288 leads to $D = -0.27$. Nosil *et*

al. (2018) reported frequencies of the striped morph of *Timema cristinae* among all green morphs (striped or unstriped), across host plants over 18 years. We used these frequencies (computed anew from the dryad repository associated with the paper) to fit a linear model for the relationship between change in frequency Δp and frequency p. We did the same for the datasets from le Rouzic *et al.* (2015), also including a fixed effect for the population of origin on the intercept.

Literature cited

Altenberg, L. 1991. Chaos from linear frequency-dependent selection. Am. Nat., doi: 10.1086/285204.

Cockerham, C. C., P. M. Burrows, S. S. Young, and T. Prout. 2015. Frequency-Dependent Selection in Randomly Mating Populations. https://doi-org.inee.bib.cnrs.fr/10.1086/282790 106:493–515. University of Chicago Press .

Gavrilets, S., and A. Hastings. 1995. Intermittency and transient chaos from simple frequency-dependent selection. Proc. R. Soc. B Biol. Sci. 261:233–238.

Haldane, J. B. S., and S. D. Jayakar. 1963. Polymorphism Due to Selection of Varying Direction. J. Genet. 58:237–242.

Nosil, P., R. Villoutreix, C. F. de Carvalho, T. E. Farkas, V. Soria-Carrasco, J. L. Feder, B. J. Crespi, and Z. Gompert. 2018. Natural selection and the predictability of evolution in Timema stick insects. Science 359:765–770.

Rice, S. H. 2004. Evolutionary theory: mathematical and conceptual foundations. Sinauer, Sunderland, Massachussets.

Wright, S., and T. Dobzhansky. 1946. Genetics of Natural Populations. Xii. Experimental Reproduction of Some of the Changes Caused by Natural Selection in Certain Populations of Drosophila Pseudoobscura. Genetics 31:125. Oxford University Press.
